# Prognostic significance of high *GFI1* expression in AML of normal karyotype and its association with a FLT3-ITD signature

**DOI:** 10.1038/s41598-017-11718-8

**Published:** 2017-09-11

**Authors:** Giacomo Volpe, David S. Walton, David E. Grainger, Carl Ward, Pierre Cauchy, Daniel Blakemore, Daniel J. L. Coleman, Peter N. Cockerill, Paloma Garcia, Jon Frampton

**Affiliations:** Institute of Cancer and Genomic Sciences, College of Medical and Dental Sciences, University of Birmingham, Birmingham, B15 2TT UK

## Abstract

Growth Factor Independence 1 (GFI1) is a transcriptional repressor that plays a critical role during both myeloid and lymphoid haematopoietic lineage commitment. Several studies have demonstrated the involvement of GFI1 in haematological malignancies and have suggested that low expression of *GFI1* is a negative indicator of disease progression for both myelodysplastic syndromes (MDS) and acute myeloid leukaemia (AML). In this study, we have stratified AML patients into those defined as having a normal karyotype (CN-AML). Unlike the overall pattern in AML, those patients with CN-AML have a poorer survival rate when *GFI1* expression is high. In this group, high *GFI1* expression is paralleled by higher *FLT3* expression, and, even when the *FLT3* gene is not mutated, exhibit a FLT3-ITD signature of gene expression. Knock-down of *GFI1* expression in the human AML Fujioka cell line led to a decrease in the level of *FLT3* RNA and protein and to the down regulation of FLT3-ITD signature genes, thus linking two major prognostic indicators for AML.

## Introduction

The concept of disease stratification promises to provide great improvements in the diagnosis, prognosis, and treatment of cancer, but requires robust and readily measurable biomarkers in order to be feasible. In the case of haematological malignancies, much has been possible through traditional phenotypic categorisation and the identification of associated genetic mutations. More recently, genome wide approaches, both at the level of gene mutations and transcriptome analysis, have enabled refinement of the classification of haematological malignancies, but there is still a great need to identify reliable molecular differences not only as prognostic indicators but also as potential therapeutic targets.

Acute myeloid leukaemia (AML) is a malignant myeloproliferative disease of the bone marrow accounting for ~10% of all haematological disorders^1, 2^. Currently, risk classification of AML with normal karyotype, representing 50% of the cases, is based on molecular biomarkers including mutations in the genes encoding FLT3, NPM1, NRAS, KRAS, KIT and CEBPA^3^. FLT3 is a tyrosine kinase receptor that is expressed in early haematopoietic cells and is essential for their proliferation and differentiation^4, 5^. *FLT3* is known to be expressed at elevated levels on the AML blast cells in over 70% of AML patients and it is among the most commonly mutated genes in AML^6^. The mutations are typically internal tandem duplications within the juxta-membrane domain of the receptor (FLT3-ITD), occurring in 20–30% of AML cases^7, 8^. FLT3-ITD mutations lead to constitutive signalling and factor-independent cell survival and proliferation^9^, and are associated with adverse clinical outcome. The leukaemogenic effects of the FLT3-ITD mutations are reflected in a distinct gene regulation signature, which is characterised by up and down regulation of DNase I hypersensitive sites and genes encoding key haematopoietic regulators and functional proteins^10^.

Growth Factor Independence 1 (GFI1), a zinc-finger transcriptional repressor that plays several critical roles in haematopoietic lineage commitment and development^11–13^ is being increasingly associated with haematological malignancies^14–18^. Mutations in *GFI1* that lead to a dominant-negative loss of function have been reported in a number of patients with congenital neutropenia^16^, whilst investigation of single nucleotide polymorphisms (SNP) associated with an increased predisposition towards AML led to the identification of a serine-asparagine substitution in the N-terminal region of GFI1 (GFI1^36N^)^17^. In line with these findings, Hönes *et al*. analysed a cohort of 524 de novo AML cases and concluded that low expression of *GFI1* is linked with an inferior prognosis^18^.

In this study we have further analysed publicly available AML data with respect to the link between *GFI1* expression and prognosis, but have stratified patients on the basis of the mutational status of their disease. This analysis has revealed that in cytogenetically normal AML (CN-AML) patients, high *GFI1* expression predicts a significantly inferior overall survival. This higher *GFI1* expression correlated with higher *FLT3* levels and a gene expression profile reminiscent of that seen in AML with the FLT3-ITD mutation, thus suggesting an important molecular connection between these factors in CN-AML.

## Results

### CN-AML patients with high *GFI1* expression have a worse clinical outcome

The analysis of AML gene profiling array data presented by Hönes *et al*.^18^ focused on the importance of low *GFI1* expression as a marker of inferior outcome in AML/MDS patients. The authors of the latter study considered all patients in the cohort reported by Verhaak *et al*.^19^, encompassing a broad spectrum of mutations including gene fusions with core transcriptional activators or repressors. We were interested to know how patients with CN-AML might represent a distinct subset in terms of the mechanisms underpinning leukaemogenesis, in particular in relation to the importance of *GFI1* and *FLT3* expression, and FLT3 mutational status.

**Figure 1:**
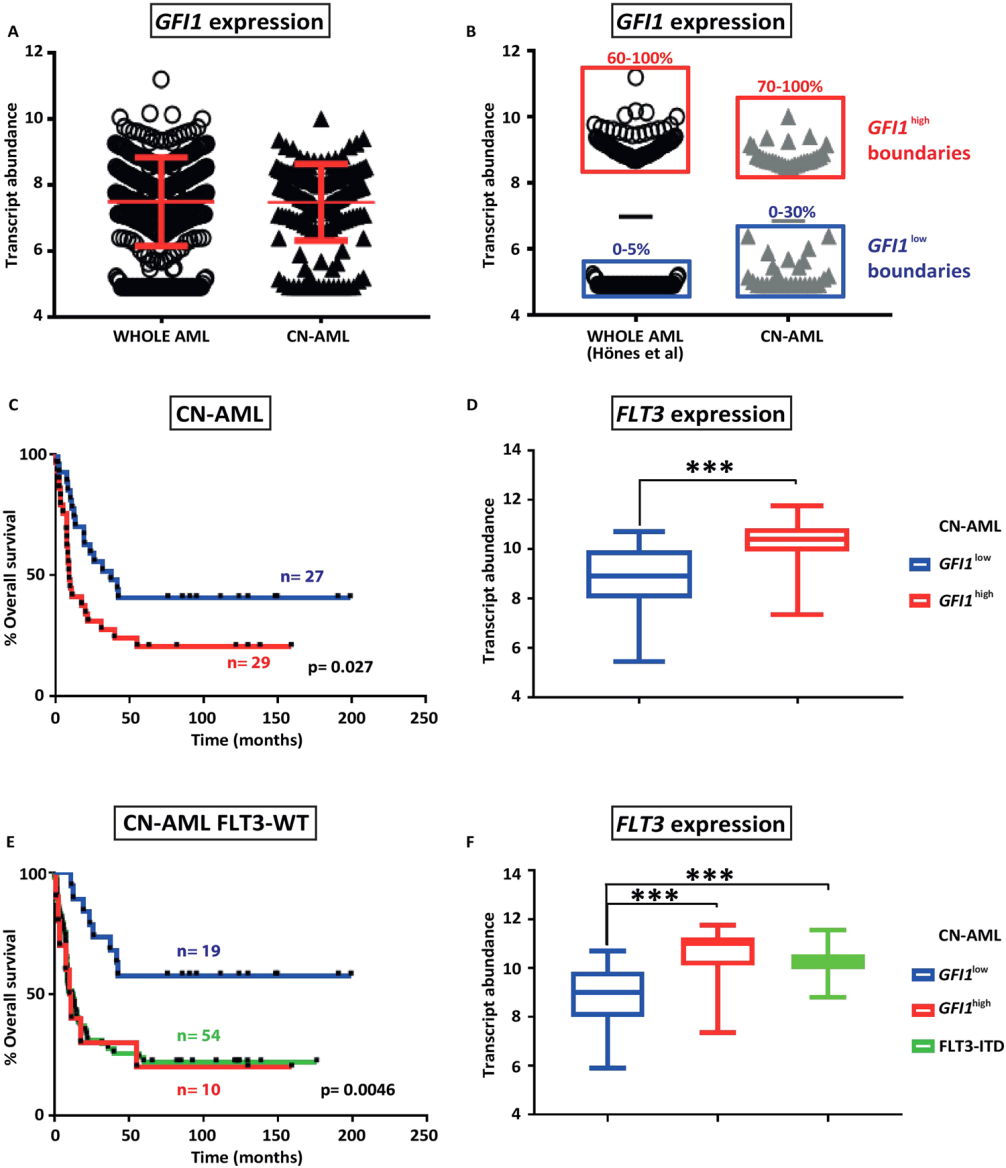
High *GFI1* expression associates with raised FLT3 expression and inferior survival in CN-AML patients. (**A**) Scatter plot representation of GFI1 expression for patient samples from the whole Verhaak *et al*.^19^ dataset (WHOLE AML) or selected on the basis of having a normal karyotype (CN-AML). (**B**) Representation of *GFI1* ^low^ and *GFI1* ^high^ expression boundaries as reported by Hönes *et al*.^18^ (0–5% low expression, 60–100% high expression) and in the present study (0–30% low expression, 70–100% high expression). (**C**) Kaplan-Meyer representation of overall survival for CN-AML patients from the Verhaak *et al*.^19^ dataset subdivided in low and high *GFI1* expressers. (**D**) Box plot representing *FLT3* transcript levels in *GFI1* ^*low*^ versus *GFI1* ^*high*^ CN-AML patient samples. Statistical significance: ***< 0.001, **< 0.01, *< 0.05. (**E**) Kaplan-Meyer estimates of overall survival for *GFI1* ^*low*^ and *GFI1* ^*high*^ FLT3-WT CN-AML samples in comparison with FLT3-ITD CN-AML patients from the Verhaak *et al*.^19^ dataset. (**F**) Boxplot depicting *FLT3* transcript levels in *GFI1* ^*low*^ and *GFI1* ^*high*^ FLT3-WT CN-AML in comparison with FLT3-ITD CN-AML samples.

For our analysis, we took the same data set as described by Verhaak *et al*.^19^ and compared survival outcomes for the whole cohort with those for patients with CN-AML, distinguishing patients with low or high expression of *GFI1*. Our CN-AML category (n = 178) excluded patients displaying abnormal or unknown karyotype or classified in the M3 FAB subgroup, as those patients are treated differently. The distribution of *GFI1* expression amongst all AML patients is not significantly different to that seen in the CN-AML subgroup (Fig. 1A). From the CN-AML group we selected the bottom 30% of the whole GFI1 expression range as low expressers (*GFI1* ^*low*^, n = 27) and the top 30% as high expressers (*GFI1* ^*high*^, n = 29), which closely encompasses the range of *GFI1* expression surveyed by Hönes *et al*.^18^ (Fig. 1B). In this cohort, *GFI1* ^*low*^ patients demonstrated a significantly inferior outcome compared to the *GFI1* ^*high*^ patients (p = 0.027) (Fig. 1C).

Similar to the observations of Hönes *et al*.^18^ when considering the total AML cohort, analysis of the mutational status of our CN-AML subgroups revealed that FLT3-ITD and NPM1c mutations were more common in *GFI1* ^*high*^ samples, these being found in 59% (p = 0.0001) and 52% (p = 0.079) of patients, when compared to the *GFI1* ^*low*^ subgroup in which they were found in 11.2% and 29% of the patients, respectively (Table 1). No statistically significant difference was observed for *IDH1, IDH2, NRAS, KRAS, CEBPA* and *EVI1* expression. Since the FLT3-ITD mutation is generally associated with higher *FLT3* expression, we next analysed the abundance of *FLT3* transcript and observed this to be higher in *GFI1* ^*high*^ samples (p = 0.0002, Fig. 1D). No differences between *GFI1* ^*low*^ and *GFI1* ^*high*^ groups were seen with respect to FAB classification, age, or sex (Table 1). Considering that FLT3-ITD is the poorest prognostic marker in CN-AML, we speculated that this could account for the inferior survival observed in the *GFI1* ^*high*^ patient subgroup. We therefore sub-classified the *GFI1* ^*high*^ CN-AML patients according to the presence or absence of FLT3-ITD. Surprisingly, *GFI1* ^*high*^ FLT3-WT patients (n = 10) still displayed lower overall survival (p = 0.0046) (Fig. 1E) and higher *FLT3* expression (p = 0.000033) that is comparable to the levels seen in patients carrying FLT3-ITD mutations (Fig. 1F). Similar to the sub-cohort of CN-AML patients carrying FLT3-ITD mutations, no differences in the FAB classification, age or sex were observed.

**Table 1:** Genetic and phenotypic characteristics of the GFI1^low^ and GFI1^high^ leukaemias in the CN-AML category.

	GFI1^low^ (n = 27)	GFI1^high^ (n = 29)	p-value
Mutations			
IDH1	1	4	0.1990
IDH2	3	2	0.4653
NPM1	8	15	0.0792
FLT3-ITD	3	17	0.0002
NRAS	3	1	0.2787
KRAS	2	0	0.2453
EVI1	2	0	0.2543
CEBPA	4	1	0.1544
FAB Classification			
M0	1	2	1
M1	7	10	0.56
M2	6	3	0.2884
M4	4	4	1
M5	7	10	0.5678
M6	2	0	0.2279
Sex			0.391
Male	12	14	
Female	15	15	
Median age	48.33	46.27	

The table shows data relating to the 56 patient AMLs included in the CN-AML sub group, including the occurrence of common specific leukaemia-associated mutations, the FAB categorisation, and the sex and age of the patients.

### High *GFI1* expression corresponds with a FLT3-ITD gene expression signature in FLT3-WT CN-AML

It was recently reported that FLT3-ITD is associated with a distinct gene expression profile, the specific signature being defined as those genes expressed at least one log_2_-fold higher in FLT3-ITD AML compared to CD34^+^ peripheral blood stem cells (PBSC), excluding those genes linked to mature myeloid differentiation that are expressed in CD14^+^ bone marrow cells at a level twice as high in PBSC^10^. This FLT3-ITD AML gene expression signature incorporates 134 genes, which were further validated against the dataset from Verhaak *et al*.^19^.

**Figure 2:**
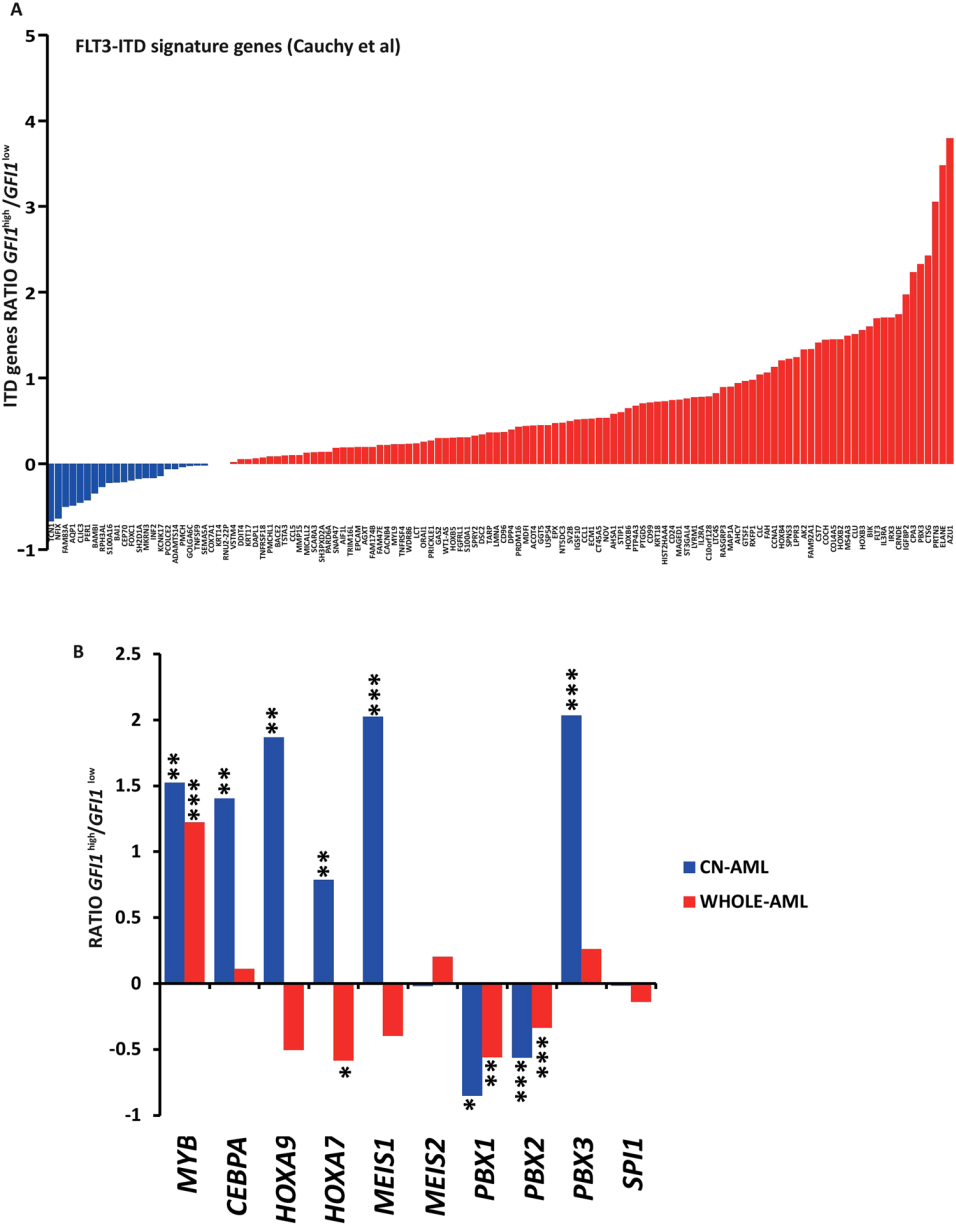
Analysis of FLT3-ITD signature genes and known FLT3 regulators in CN-AML (**A**) Histogram representing gene expression ratio of FLT3-ITD molecular signature in *GFI* ^*high*^ versus *GFI1* ^*low*^ FLT3-WT CN-AML patient samples from the Verhaak dataset. (**B**) Analysis of expression levels of known regulators of FLT3 activity in leukaemia, comparing *GFI1* ^*low*^ versus *GFI1* ^*high*^ FLT3-WT CN-AML samples (blue bars) or WHOLE AML samples (red bars). Statistical significance: ***< 0.001, **< 0.01, *< 0.05

We therefore investigated whether high *GFI1* expression in the CN-AML FLT3-WT leukaemias corresponds to higher abundance of the FLT3-ITD molecular signature genes. Using the AML gene expression dataset of Verhaak *et al*.^19^, we determined the expression of the FLT3-ITD signature genes in FLT3-WT CN-AML patients and stratified these according to the level of *GFI1* RNA. Strikingly, this analysis revealed that in *GFI1* ^*high*^ AML the expression of genes making up the FLT3-ITD signature was largely elevated compared to AML with low *GFI1* expression (Fig. 2A). Further to validate these findings, we confirmed the association between high *GFI1* levels and FLT3-ITD signature genes in another independent CN-AML cohort from a study performed on 251 CN-AML samples, reported by Kohlmann *et al*.^20^ (Fig. 1).

### Known regulators of FLT3 are over expressed in *GFI1*^*high*^ CN-AML

Recent reports have demonstrated a mechanism for the Gfi1-dependent acceleration of K-Ras driven myeloproliferative disorders in mice through the over expression of *HoxA9* and other AML-related genes^15, 21^, eventually leading to the development of AML. This study also revealed elevated expression of the HoxA9 partner proteins Meis1 and Pbx1, and of their known target genes *Myb* and *Spi1/PU.1*. Considering that these transcription factors, together with CEBPα, are among the main regulators of FLT3 activity in AML^22–24^, we next sought to determine whether high *GFI1* levels are associated with differences in their expression in CN-AML patients. Comparing *GFI1* ^*high*^ versus *GFI1* ^*low*^ FLT3-WT CN-AMLs, we found higher expression in the former of *MYB* (p = 0.0029), *CEBPA* (p = 0.0043), *HOXA9* (p = 0.0052), *HOXA7* (p = 0.0033), *MEIS1* (p = 0.000043) and *PBX3* (p = 0.0001), while lower abundance was observed for *PBX1* (p = 0.011) and *PBX2* (p = 0.00004). No differences were observed in the levels of *MEIS2* and *PU.1* (Fig. 2B). The latter is unsurprising as we have previously shown that *PU.1* is directly involved in regulating *FLT3* in haematopoietic progenitor cells but not in leukaemic cells^23–25^. While the lower levels of *PBX1* and *PBX2* observed in high *GFI1* patients are in agreement with the findings presented by Horman *et al*.^15^, higher expression of *HOXA9* and its partner *MEIS1* are in contrast with their observations. This difference could be explained by the fact that our analysis was performed in CN-AML patients only, therefore excluding patients harbouring chromosomal translocations, such as those involving *MLL*, in which *HOXA9* expression is often deregulated^26, 27^. To address this, we performed the same analysis using patient samples from the whole AML cohort, classified into *GFI1* low and high expressers. In line with previous observations, this analysis revealed lower expression levels of *HOXA9*, *HOXA7* and *MEIS1* (Fig. 2B).

### Reduction of *GFI1* in AML cell lines leads to decreased expression of FLT3-ITD signature genes

**Figure 3:**
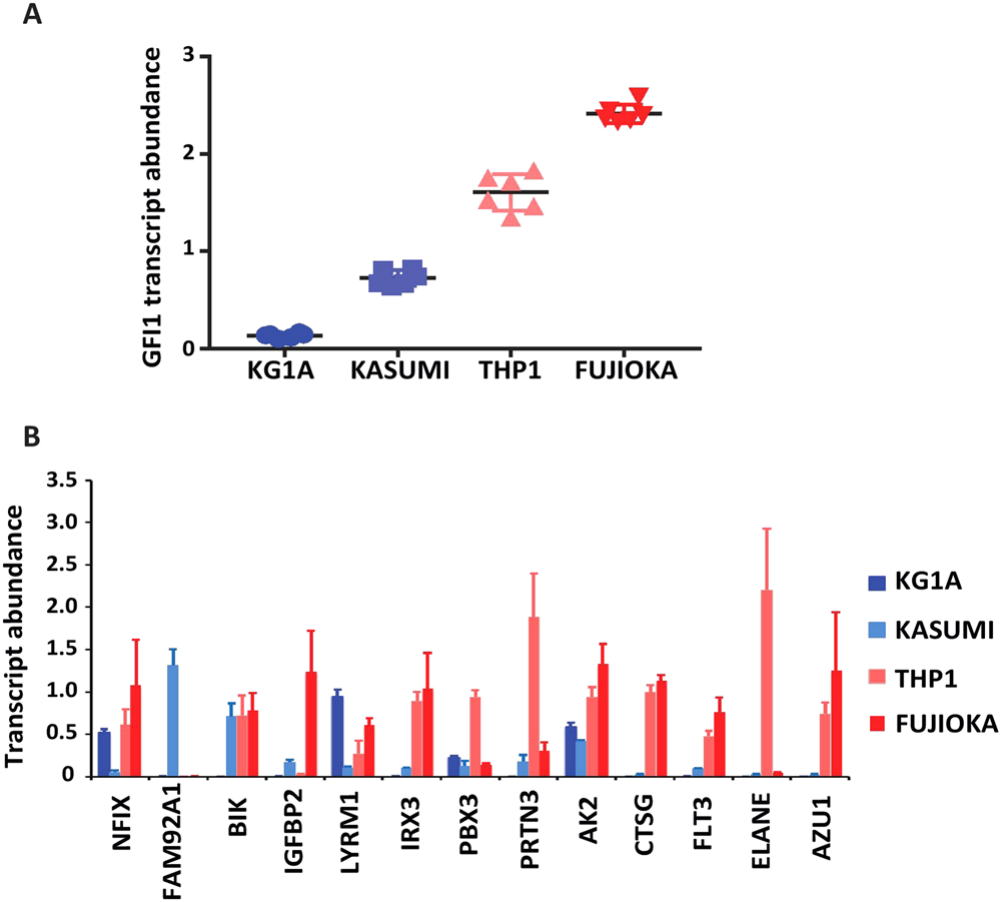
Expression analysis of a set of FLT3-ITD molecular signature genes in human AML cell lines (**A**) Scatter plot showing the relative abundance of *GFI1* transcript in KG1A, Kasumi-1, THP1 and Fujioka human AML cell lines. (**B**) Histogram depicting the relative transcript abundance of FLT3-ITD signature component in the Fujioka cell line.

In order to assess the significance of our observed correlation between *GFI1* expression and the level of FLT3-ITD signature genes in FLT3-WT CN-AML, we next investigated whether manipulation of *GFI1* expression in a human AML cell line would lead to corresponding changes in the expression of the FLT3-ITD signature genes. First, we measured the relative expression of *GFI1* RNA in human AML cell lines characterised by the expression of wild type FLT3 receptor, including KG1a, Kasumi-1, THP1, and Fujioka. Quantitative RT-PCR revealed that *GFI1* is least expressed in KG1a and most highly expressed in Fujioka cells (Fig. 3A). To determine if higher expression of the FLT3-ITD signature genes also correlates with *GFI1* level in these cells lines, we then tested the transcript abundance of the FLT3-ITD signature genes *NFIX, FAM92A1, BIK, IGFBP2, LYRM1, IRX3, PBX3, PRTN3, AK2, CTSG, FLT3, ELANE* and *AZU1*. Interestingly, this analysis demonstrated higher expression of most of these genes, with the exception of *FAM92A1*, in those cell lines with the highest expression of *GFI1* (Fig. 3B).

**Figure 4:**
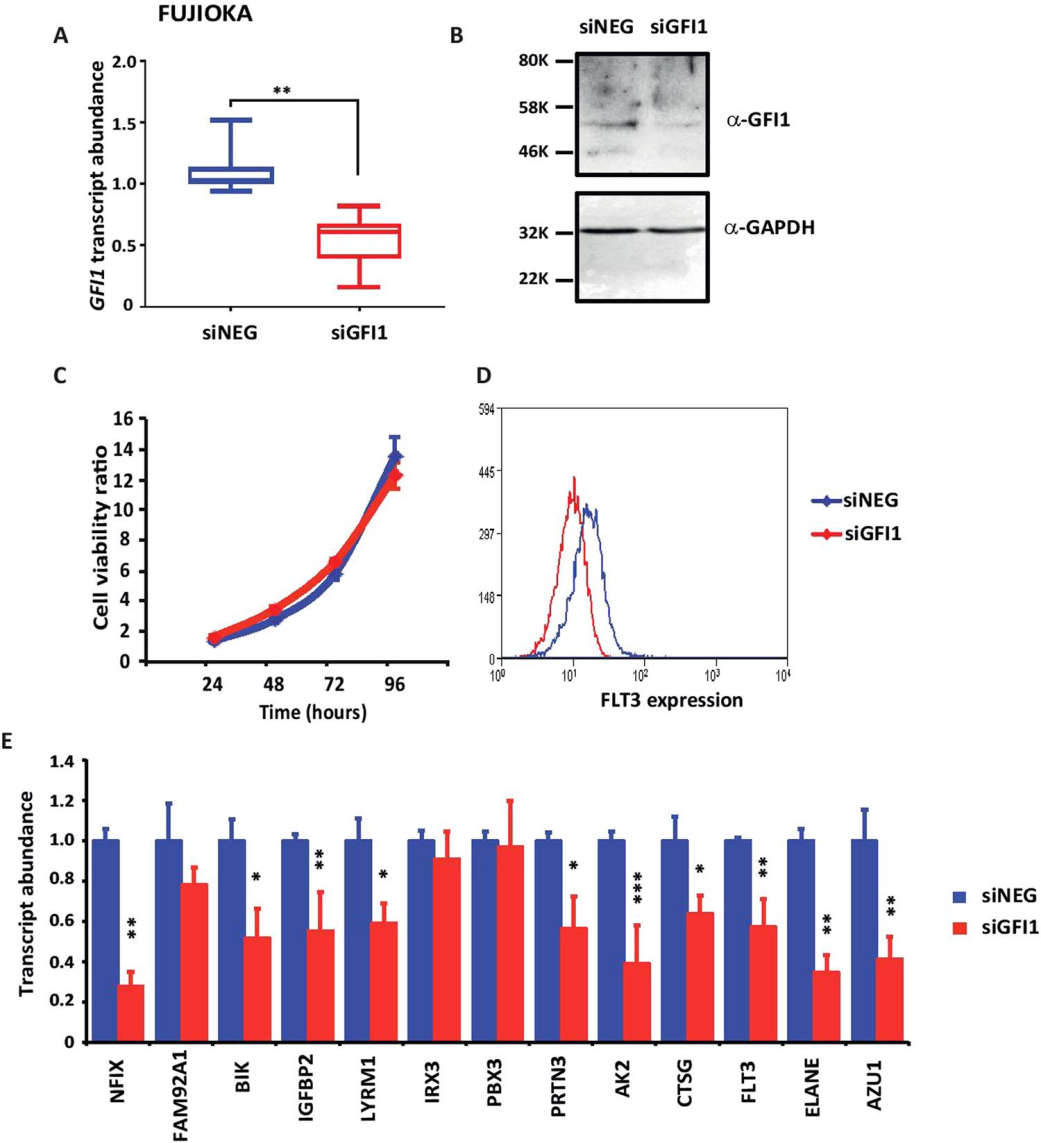
siRNA-mediated downregulation of *GFI1* expression in Fujioka cells (**A**) *GFI1* transcript abundance was determined by quantitative-PCR in cells transfected with *GFI1* siRNA and negative control siRNA. Results are representative of 5 independent experiments. Statistical significance was calculated using student’s t-test. ***< 0.001, **< 0.01, *< 0.05. (**B**) Immunoblot analysis of GFI1 protein in extracts from cells transfected with *GFI1* siRNA and negative control siRNA. GAPDH was used as internal control. For detection of GFI1 the membrane was cut into two just below the 46 KDa ladder mark to avoid detection of a cross-reacting non-specific band at around 40 KDa. (**C**) Cell viability was calculated by counting transfected cells every 24 hours for 4 consecutive days. (**D**) Flow cytometric staining of transfected Fujioka cells with a labelled antibody against FLT3. (**E**) RNA quantification by q-PCR of FLT3-ITD molecular signature component 24 hours post siRNA transfection in Fujioka cells. Statistical significance was calculated using student’s t-test. ***< 0.001, **< 0.01, *< 0.05.

Next, we performed siRNA-mediated knockdown of *GFI1* in the Fujioka line to determine if the highlighted FLT3-ITD signature genes were dependent on the high level of *GFI1* expression in these cells. Fujioka cells were electroporated with siRNA targeting either *GFI1* or a scrambled negative control. Cells were harvested 24 hours post transfection to assess *GFI1* expression levels by quantitative RT-PCR, revealing a highly significant down regulation of both *GFI1* RNA (p < 0.001) (Fig. 4A). Cells were additionally collected at 48 hours for preparation of protein extracts and assessment of GFI1 protein levels by immunoblotting (Fig. 4B). Cell counts performed every 24 hours post transfection revealed that *GFI1* down regulation had no significant effect on cell growth (Fig. 4C). Fujioka cells were maintained in culture for 96 hours post transfection at which point the expression of several surface markers associated with myeloid differentiation was tested by immunofluorescence / flow cytometry. CD34, CD38, CD11b, CD14, and CD56 showed no measurable differences (data not shown), whereas FLT3 surface expression was significantly decreased (Fig. 4D). Importantly, analysis of the expression of FLT3-ITD signature genes demonstrated a reduction in the abundance of several of these, the most notable being *NFIX, BIK, IGFBP2, LYRM1 PRTN3, AK2, CTSG, FLT3, ELANE*, and *AZU1* (Fig. 4E).

## Discussion

In this study, we show that higher levels of *GFI1* expression can be used to predict unfavourable outcome in AML patients with normal cytogenetics, and thereby provide a possible stratification for therapy choices. Additionally, we found that higher *GFI1* levels are also associated with higher *FLT3* expression and elevated levels of FLT3-ITD signature genes even in patients with non-mutated *FLT3*.

Compared to earlier findings by other groups, the way in which we have distinguished between CN-AML and AML with abnormal cytogenetics illustrates how stratification based on a single factor such as *GFI1* expression can lead to quite different conclusions. Hence, Hönes *et al*.^18^ sub-fractioned AML patients purely according to *GFI1* expression revealing that overall inferior survival associated with low *GFI1* levels. In the present study we first classified AML based on karyotype, dividing them into abnormal karyotype only and CN-AML only and then sub-fractioning these two groups into low and high *GFI1* expressers. In the first group we observed that low *GFI1* expressers were indeed associated with inferior outcome, in agreement with findings reported previously. Strikingly, when the same criterion was applied to the CN-AML group, we observed that high *GFI1* expressers were instead characterized by worse overall survival.

We also considered how the status of FLT3 expression or mutation might correlate with the stratification. In their study of unfractionated AML Hönes *et al*.^18^ suggested that FLT3-ITD associates with high *GFI1* levels and favourable outcome measures, which is a surprising finding given that FLT3-ITD is the most significant prognostic factor in AML and is associated with unfavourable outcome. However, as we have discussed, patients from this cohort were not subcategorized based on karyotype, raising the possibility that GFI1 impacts on leukaemia in different ways according to the driver mutations. When we looked at the CN-AMLs we also observed an association of FLT3-ITD with the *GFI1* ^*high*^ group, suggesting that FLT3-ITD was responsible for the inferior outcome observed in those patients. To test this, we looked instead at only those CN-AML that had a wild type FLT3 status, again classifying into *GFI1* ^*low*^ versus *GFI1* ^*high*^. This analysis showed that the overall survival of the *GFI1* ^*high*^ AML was much worse, and moreover, these AML exhibited a significantly higher expression of *FLT3*.

Several studies, mainly in mouse models, have addressed the role of GFI1 in haematological malignancies. In line with an association between high *GFI1* expression and poor outcomes it has been reported that *Gfi1* cooperates with *Pim-1* and *Myc* in the genesis of T-cell lymphoma^28^. High levels of *GFI1* expression were also found to be important in accelerating T-cell proliferation and preventing induced cell death in Jurkat T-cells^29^. Furthermore, Khandanpour *et al*.^21^ reported a requirement for Gfi1 in the establishment and progression of murine B-cell lymphoma and T-cell acute lymphoblastic leukaemia driven by various genetic lesions, the ablation of *Gfi1* leading to significant tumour regression and increased host survival in a p53-dependent manner^30^. In apparent contrast to these latter findings, the role of Gfi1 in the regulation of HoxA9, Meis1 and Pbx1 in murine myeloid cells appears to prevent predisposition to haematological malignancies, its loss-of-function decreasing the latency of KRas-driven MDS^15^. These observations were further supported by the identification of a SNP in the *GFI1* gene that generates a variant protein (GFI1^[36N]^), which experimentally accelerated KRas-driven myeloproliferative disorders by inducing epigenetic changes at the HoxA9 locus^21^. Similarly, studies using a humanized *GFI1* knockdown mouse model showed that lower *GFI1* levels accelerate the progression of MLL-AF9- and NUP98-HoxD13-driven AML.

These contrasting observations from different model systems clearly imply that the functional consequences of GFI1 activity can be quite different and context dependent, and there is a need to investigate more broadly what mechanisms operate. For example, one could speculate that in AML involving translocations in major chromatin remodellers, such as in MLL-AF4 gene fusions, high levels of *GFI1* might antagonise runaway activation while the converse would fail to compensate the latter, resulting in additionally impaired differentiation and thus contributing towards more aggressive leukaemogenesis. In CN-AML however, *GFI1* might play a more physiological role with higher expression corresponding to repression of alternative lineages, thus favouring differentiation and thus more manageable AML.

To gain an initial insight into the influence of high *GFI1* expression in FLT3-WT CN-AML we sought to determine if there is any correspondence with the signature of gene expression that characterises FLT3-ITD AML. Interestingly, this analysis showed that, despite those samples having wild type FLT3, the molecular signature of FLT3-ITD was prominent in *GFI1* ^*high*^ cells. More importantly, in the attempt to validate the relevance of these findings, we observed that a number of the components of the FLT3-ITD molecular signature (*IGFBP2, PRTN3, AK2, CTSG, ELANE* and *AZU1*) were down regulated after siRNA-mediated silencing of *GFI1* in Fujioka human AML cell line. This suggests that one of the main mechanisms of *GFI1* leading to inferior outcome might directly or indirectly act through these genes.

Taken together, our results call for a more elaborate stratification of AML patients to ensure proper diagnosis and effective treatment and demonstrate that high GFI1 expression is a reliable and powerful prognostic indicator for CN-AML. Our findings also suggest that it will be fruitful to investigate in detail how GFI1 is linked to driver mutations, including those involving FLT3.

## Materials and Methods

### Patient profiling arrays

Log_2_ transformed, MASS normalised microarray expression data from Verhaak *et al*.^19^ and Kohlmann *et al*.^20^ were retrieved from GEO accession number GSE6891 and GSE15434, respectively. CN-AML patients reported in those cohorts were ranked according to their *GFI1* expression into *GFI1* ^*high*^ (top 30% of expression range) and *GFI1* ^*low*^ (bottom 30%). Gene expression fold changes were expressed as log_2_ ratios of *GFI* ^*high*^ */GFI1* ^*low*^ gene expression levels. Up- and down-regulated genes were identified as those displaying log_2_ fold change ≥ 1 and p < 0.05. For data from Cauchy *et al*.^10^, and gene expression levels and fold changes from Verhaak *et al*.^19^ and Kohlmann *et al*.^20^ were retrieved for the previously published 134-gene FLT3-ITD signature.

### AML cell lines

KG1A, Kasumi-1, THP1, and Fujioka human AML cell lines were grown in RPMI 1640 medium supplemented with 10% foetal bovine serum, 50 u/ml penicillin, 50 µg/ml streptomycin, and 2mM L-Glutamine. Cells were maintained at 0.5 × 10^6^ cells/ml and were washed with phosphate buffered saline solution between passages.

### Transfections, cell viability assays, and flow cytometry analysis

In total, 5 × 10^6^ Fujioka cells were electroporated with 300 nM of either GFI1 siRNA (s199938, Ambion – Life Technologies) or scrambled control siRNA (4390843 Silencer Select Negative Control #1) using the BIORAD Gene Pulser XCell (BIORAD, Hercules, California, US). Cell proliferation assays were performed in triplicate with Fujioka cells transfected with *GFI1* siRNA and negative control. Cells were counted every 24 hours for 4 consecutive days and growth curves were obtained by quantifying the number of viable cells. Flow cytometry analysis was performed on transfected Fujioka cells stained using antibodies against CD34, CD38, CD11b, CD14, CD56, and FLT3. All the antibodies were purchased from eBioscience.

### Quantitative PCR and Western Blot

RNA extraction was performed 24 hours post siRNA transfection using RNeasy Mini kit (Qiagen) and first-strand cDNA synthesis was performed using standard protocols. Quantitative RT-PCR analysis of *GFI1, FLT3, GAPDH*, and the components of the FLT3-ITD molecular signature was performed using predesigned Taqman gene expression assays (Applied Biosystems). Total proteins obtained from Fujioka cells transfected with either GFI1 siRNA or scrambled control were used for Western Blot analysis. Antibodies were as follows: anti-GFI1 mouse monoclonal (1:500, Santa Cruz Biotechnology) and anti-GAPDH mouse monoclonal (1:10000 dilution, Abcam).

### Data availability

All patient data described were obtained from publically available databases.

## Electronic supplementary material

Figure S1
