# Supplementary material for: Prognostic significance of high GFI1 expression in AML of normal karyotype and its association with a FLT3-ITD signature
Source: Sci Rep. 2017 Sep 11;7:11148. doi: 10.1038/s41598-017-11718-8 (PMC5593973; doi:10.1038/s41598-017-11718-8)
Supplement: Supplementary file 1 — Figure S1 [file 41598_2017_11718_MOESM1_ESM.pdf]

Supplementary Information

Prognostic significance of high GFI1 expression in AML of normal karyotype and its association with a FLT3-ITD signature

Giacomo Volpe, David S Walton, David E Grainger, Carl Ward, Pierre Cauchy, Daniel Blakemore, Daniel J L Coleman, Peter N Cockerill, Paloma Garcia and Jon Frampton.

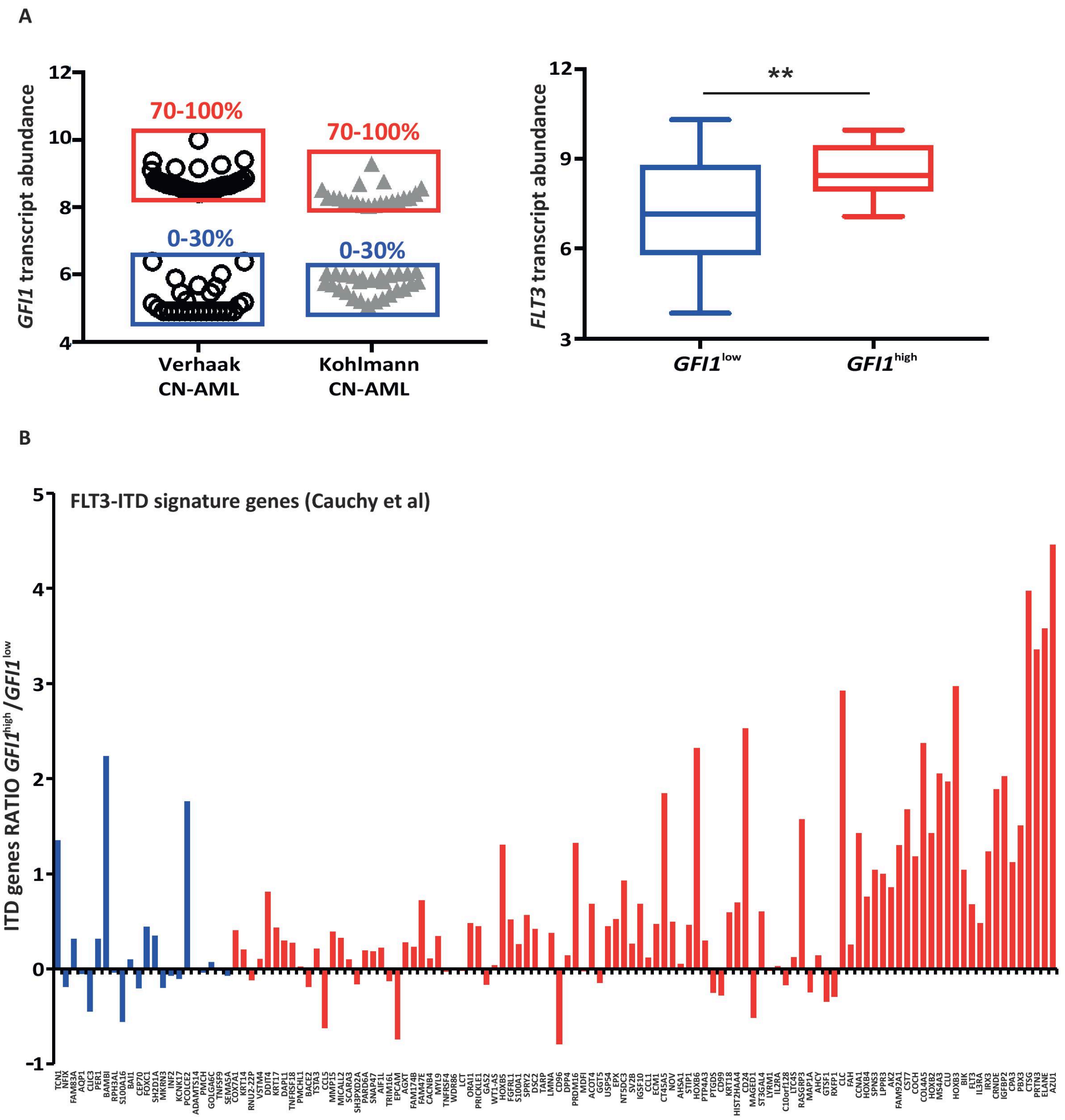

Figure S1. Analysis of FLT3-ITD genes in an independent CN-AML dataset

(A) Representation of the 0-30% *GFI1*^{low} and 70-100% *GFI1*^{high} expression boundaries comparing samples from the Verhaak et al[19] and the Kohlmann et al datasets. Boxplots in the right panel represent the abundance of the *FLT3* transcript in *GFI1*^{low} versus *GFI1*^{high} CN-AML patient samples from the Kohlmann dataset. (B) Histogram representing expression of FLT3-ITD molecular signature genes in *GFI*^{high} versus *GFI1*^{low} FLT3-WT CN-AML patient samples from the Kohlmann dataset. The ranking of the genes is the same as that depicted in FIG2A.
